# Automated Estimation of Crop Yield Using Artificial Intelligence and Remote Sensing Technologies

**DOI:** 10.3390/bioengineering10020125

**Published:** 2023-01-17

**Authors:** Qazi Mudassar Ilyas, Muneer Ahmad, Abid Mehmood

**Affiliations:** 1 Department of Information Systems, College of Computer Sciences and Information Technology, King Faisal University, Al Ahsa 31982, Saudi Arabia; 2 Endicott College of International Studies, Woosong University, Daejeon 34606, Republic of Korea; 3 Department of Management Information Systems, College of Business Administration, King Faisal University, Al Ahsa 31982, Saudi Arabia

**Keywords:** precision agriculture, sensory images, data augmentation, feature extraction, deep learning, data analysis

## Abstract

Agriculture is the backbone of any country, and plays a viable role in the total gross domestic product (GDP). Healthy and fruitful crops are of immense importance for a government to fulfill the food requirements of its inhabitants. Because of land diversities, weather conditions, geographical locations, defensive measures against diseases, and natural disasters, monitoring crops with human intervention becomes quite challenging. Conventional crop classification and yield estimation methods are ineffective under unfavorable circumstances. This research exploits modern precision agriculture tools for enhanced remote crop yield estimation, and types classification by proposing a fuzzy hybrid ensembled classification and estimation method using remote sensory data. The architecture enhances the pooled images with fuzzy neighborhood spatial filtering, scaling, flipping, shearing, and zooming. The study identifies the optimal weights of the strongest candidate classifiers for the ensembled classification method adopting the bagging strategy. We augmented the imagery datasets to achieve an unbiased classification between different crop types, including jute, maize, rice, sugarcane, and wheat. Further, we considered flaxseed, lentils, rice, sugarcane, and wheat for yield estimation on publicly available datasets provided by the Food and Agriculture Organization (FAO) of the United Nations and the Word Bank DataBank. The ensemble method outperformed the individual classification methods for crop type classification on an average of 13% and 24% compared to the highest gradient boosting and lowest decision tree methods, respectively. Similarly, we observed that the gradient boosting predictor outperformed the multivariate regressor, random forest, and decision tree regressor, with a comparatively lower mean square error value on yield years 2017 to 2021. Further, the proposed architecture supports embedded devices, where remote devices can adopt a lightweight classification algorithm, such as MobilenetV2. This can significantly reduce the processing time and overhead of a large set of pooled images.

## 1. Introduction

The United Nations estimates that the world population reached the eight billion mark on 15 November 2022 [1]. It is expected to reach 8.5 billion in 2030 and 9.7 billion in 2050. This increase in population has motivated many countries to prioritize food security in their strategic plans [2,3]. The recent global crises of the COVID-19 pandemic and the Russia–Ukraine war have further complicated the situation due to supply chain disruptions. Owing to a harsh climate in most parts of the country, a lack of fertile land, and scarce water resources, Saudi Arabia relies on imports for over 80% of its food needs [4]. Hence, it is no surprise that Saudi Vision 2030 makes food security one of its priorities [5].

The ultimate objective of food security can be achieved through several means, such as increasing arable land, reducing food wastage, using advanced technologies in agriculture, improving resource utilization, and effective planning. Planning and policy-making play a vital role in achieving food security for a country such as Saudi Arabia, which lacks the essential ingredients of agricultural produce, namely land and water. The planning for food security in Saudi Arabia involves reducing food waste and optimizing indigenous growth to reduce reliance on imports. Several initiatives have been proposed, and the government is actively working to reduce food loss and waste [6,7,8]. The scope of this study is limited to the second aspect of planning, related to local agricultural produce.

Recent technological advancements, improved awareness, and reduced costs have galvanized the adoption of precision agriculture in the last few years [9]. One of the active areas of research in precision agriculture is automatic crop yield estimation using artificial intelligence and remote sensing technologies [10]. While the manual methods for crop yield estimation are laborious and unscalable, automatic estimation is cost-effective and highly efficient. With high accuracy and minimal cost, real-time crop yield estimates can help farmers and government entities manage existing supplies, plan imports, and support strategic planning for the future [11]. These techniques have proven effective for estimating field- and region-level crop yield [12].

Automatic crop yield estimation typically relies on sensory data provided by satellite or unmanned aerial vehicles. The researchers have developed several indices based on this imagery to assess vegetation in an area. At the heart of these indices is spectral reflectance measurement in visible (Red) and near-infrared (NIR) bands. Healthy vegetation reflects more solar radiation in the NIR than in the Red band [13]. In addition, vegetation stress has a direct relationship with the Red band and an inverse relationship with the NIR band. This relationship is expressed by the normalized difference vegetation index (NDVI), one of the most commonly used indices for vegetation measurement, is given in Equation (1) [13].

(1) $$NDVI=\frac{NIR-Red}{NIR+Red}$$

A higher value of NDVI shows the presence of green vegetation in an area.

The vegetation condition index (VCI) compares the current NDVI value to the respective values observed in the previous years to identify favorable vegetation conditions. A value of zero for VCI indicates the most unfavorable conditions for vegetation, while a value of 100 represents the best conditions. The VCI can be calculated by Equation (2) [13].

(2) $$VCI=\frac{NDVI-NDVI_{min}}{NDVI_{max}-NDVI_{min}}\times 100$$

Due to the limitation of NDVI, Gitelson proposed the wide dynamic range vegetation index (WDRVI) by incorporating crops’ physiological and phenological characteristics [14]. The index can be calculated using Equation (3) [14].

(3) $$WDRVI=\frac{a\times \rho_{NIR}-\rho_{Red}}{a\times \rho_{NIR}+\rho_{Red}}$$

where $\rho_{NIR}$ and $\rho_{Red}$ are values of reflectance in near-infrared and visible bands, while a is a weighing coefficient whose value ranges between 0.1 and 0.2.

We have provided a brief exposé of the primary vegetation indices. After critically reviewing over 100 vegetation indices, Xue and Su argue that these indices must be used with great caution [15]. It is also worth noting that, in addition to vegetation indices, crop yield estimation depends on a diverse set of factors, such as soil characteristics, canopy, rainfall, subsurface water, environment, and air temperature. As remote sensing technologies are widely used in all these domains, a massive amount of sensory data is collected from various resources. The primary sources of sensory data include field surveillance cameras, temperature sensors, humidity sensors, fire-alert sensors, flood-warning sensors, and weather monitoring sensors. Manual analysis and processing of such large amounts of diverse data for crop yield estimation are time-consuming, inaccurate, and prone to errors. In recent years, machine learning technologies have successfully used such data to solve prediction problems [16,17,18,19].

The current study addresses various issues involved in crop yield estimation using machine learning. First, the performance of machine learning algorithms for crop yield estimation is adversely affected by the low quality of pooled images used as input. Manual feature extraction is another issue that needs to be addressed because of its laborious nature. While many techniques focus on the tasks of crop classification and yield estimation individually, combining these tasks adds to the complexity of this study. Lastly, the limited capabilities of lightweight embedded devices used for real-time crop monitoring with remote sensors pose another challenge in the study. In the following, we briefly overview some salient works related to these issues.

The presence of noise in the form of clouds or natural aerosols in the images acquired by satellites or unmanned aerial vehicles remains an open challenge in smart farming [20]. Tsouros et al. stress the need for image quality enhancement for crop yield estimation [21]. Wang et al. reviewed various filters for image contrast enhancement [22]. Li et al. used the image fusion technique to enhance image contrast [23]. Manual feature extraction is a limitation of conventional machine learning methods [24]. Deep learning techniques are used for crop yield estimation to overcome this limitation [25,26,27]. However, a limitation of deep learning algorithms is the high computational requirements, making them unsuitable for lightweight devices. Few studies have proposed machine learning pipelines for crop classification and yield production. Meraj et al. proposed a masking technique to classify images with different crops [28] and predicted wheat yield based on the images classified as belonging to the wheat crop. Lastly, some studies have implicitly addressed the suitability of proposed models to be deployed on lightweight embedded devices for real-time monitoring [29,30,31]. Hence, there is a need to develop crop yield estimation techniques suitable for lightweight devices.

To overcome the limitations of existing work, we proposed a hybrid ensemble method to signify a variety of crops’ yield estimation and classification. The main contributions of this study are as follows.

- The proposed hybrid ensemble method is based on intensive image preprocessing inspired by fuzzy neighborhood spatial filtering, scaling, flipping, shearing, and zooming.
- Considering different use cases of convolutional neural networks (CNNs) simulated on multiple sensory data, we evaluated the performance of the visual geometry group (VGG) with defined/customized image kernels.
- Although the performance of state-of-the-art (SOTA) methods is architecture-dependent, the performance of VGG-16 was noted to be better with relatively faster training time. It ultimately helped to achieve better classification accuracy by empowering the weaker classifiers.
- The ensemble model outperforms individual classifiers with the help of the best feature extractor, VGG-16, among other convolution networks, including Inception V3, VGG-19, DeepLoc, and SqueezeNet, simulated on larger sets of sensory imaging data.
- The proposed ensemble method lays the foundation to work with embedded devices efficiently by adopting VGG-16 (in general) and MobileNetV2 (in particular) for remote sensory data.

The rest of the manuscript is organized as follows. Section 2 provides the limitations of related work. Section 3 presents the proposed methodology with a description of the essential components of the architecture. Section 4 discusses the significant results, and Section 5 concludes the research work.

## 2. Related Work

This section reviews the recent research focused on machine learning approaches combined with remote sensing technologies for crop yield prediction of various types of crops. The crop yield prediction has been carried out using traditional and deep learning-based methods. In general, the methods of the former type execute with low computational resources but provide comparatively lower performance. On the other hand, the latter kind of methods has generally achieved superior performance with high computational costs. Several studies have performed a detailed comparison of the use of each of the approaches. Oikonomidis et al. [32] presented the state-of-the-art application of deep learning in crop yield prediction. They discovered that the most frequently used algorithm, Convolutional Neural Network (CNN), performs the task best. One of the most significant issues is the lack of datasets of larger size, which increases the likelihood of overfitting and, as a result, worse model performance. Rashid et al. [33] reviewed the use of machine learning techniques in palm oil yield prediction. The study found that the most commonly used traditional machine learning models are logistic regression, random forest, and neural networks. Most of the recent works are found to be focusing on deep learning models.

Further, the study reports that because of the minimal utilization of feature sets in palm oil yield prediction, the state-of-the-art suffices for neither the selection of the best feature set nor the prediction model. Another comprehensive literature assessment identifies existing research gaps in the specific area of deep learning approaches combined with remote sensing [34]. The study aids in understanding the impact of vegetation indices and environmental conditions on crop yield. According to this study, the most extensively used deep learning algorithms for crop yield prediction are long short-term memory and convolutional neural networks. Satellite remote sensing technology is the most often utilized remote sensing technology.

Furthermore, the study suggests that vegetation indices are the most used factor for predicting agricultural productivity. However, it has been discovered that the most utilized properties in the literature may not always apply to all techniques. Table 1 provides a summary of the recent works in the domain of the current study. The focus areas listed in the table highlight the coverage of the challenges facing the machine learning approaches for crop yield estimation described in the introduction section.

### 2.1. Traditional Methods

Several approaches have appeared in the literature that address the estimation of yield for more than one crop simultaneously. However, wheat yield is most typically predicted jointly with other crops in these studies. Paudel et al. [29] proposed an approach for forecasting yield for five crops (soft wheat, spring barley, sunflower, sugar beet, and potatoes) in large-scale production scenarios. For this purpose, they coupled crop modeling agronomic principles with machine learning and provided a baseline workflow focused on correctness, modularity, and reusability. The approach enhanced the correctness by developing features based on different data sources, including crop simulation outputs, meteorological, remote sensing, and soil data from the MCYFS dataset. They provided a reusable workflow to suit diverse crops and regions. The reported case studies estimated yield at the regional levels for the five crops in three countries (the Netherlands, Germany, and France) with high accuracy. Meroni et al. [30] predicted barley, soft wheat, and durum wheat yields in Algeria. They used a suite of machine algorithms in different settings to determine the best model compared to the benchmarks set by the study. The study used public satellite and climate data from the European Commission Joint Research Center. One limitation of this study is that it relies extensively on the continuous calibration of various models, and does not determine a single model or a combination of features that can deliver consistently high performance. The problem of inconsistent forecasts across spatial levels is effectively addressed by Paudel et al. [35]. At the heart of their approach is the idea of forecasting yield at a regional level. The proposed generic workflow is used to predict the yields of six crops (wheat, barley, sunflower, grain maize, sugar beets, and potatoes) across nine European countries. The study shows that a model working on various spatial levels of regional-level data instead of the national level can provide substantially better performance.

Some studies have focused on predicting the yield of a single crop. Unsurprisingly, wheat yield prediction dominates among these studies. Zhou et al. [31] proposed a model to predict wheat yield at the national level in China. For this purpose, the study analyzed the effect of nine climate variables, along with three remote sensing-specific metrics. The prediction was carried out by adopting random forest, support vector machine, and least absolute shrinkage and selection operator. The study integrated climate and remote sensing data. In addition to obtaining higher accuracy for country-level predictions, the study has highlighted some other interesting findings. For instance, climate data from the entire growing season offered more information for yield prediction than remote sensing data.

Further, compared to remote sensing data, the additional contribution for yield prediction in winter wheat planting zones that benefited from climate data declined from sowing to maturity. In a similar study, climate records and satellite image time-series data were used to predict wheat yields in Australia [36]. The study adopted nine base learners and two ensembles to train on high-resolution yield maps, NDVI time series data, and climate records comprising rainfall and maximum temperature data.

The predictions made by non-linear models were more accurate than those of linear models. At the same time, support vector regression with radial base functions outperformed the other models in making pixel-level predictions. Furthermore, ensemble approaches did not indicate a substantial advantage over the single best model in the study’s specific setting. Bian et al. [37] used multi-spectral remote sensing data from UAV platforms to develop a field-level prediction model for wheat crops in China. They extracted ten vegetarian indices from images of wheat acquired from a UAV at five different growth stages and used them for tuning the model variables of six other machine learning models. The study compared the prediction results of the adopted models between the single and multiple growth stages. They found Gaussian process regression to outperform the other models in both settings. However, they have not compared the performance of their proposed model with the other existing works in the domain that work on similar data. A hybrid approach was also developed to forecast wheat yield in China that couples machine learning and a global dynamical atmospheric prediction system [38]. The approach used three machine algorithms, i.e., XGBoost, random forest, and support vector regression, along with the multiple regression model. Four types of data were utilized: crop yield data, satellite data, observational climate data, and atmospheric prediction data. sThe study discovered that XGBoost outperforms all other models when trained on atmospheric forecast data.

Some works have conducted acreage classification and yield estimation simultaneously. Meraj et al. [28] first used random forest and support vector machine classifiers to perform a supervised classification aimed at acreage assessment for wheat in India. Then, they used the Carnegie–Ames–Stanford Approach (CASA) model to estimate the wheat crop. Later, the estimation results of CASA were verified using 30 observational points. Barley is one of the world’s strategic agricultural products, and its yield prediction is critical for guaranteeing food security. Sharifi [39] integrated remote sensing and climate data to build a machine learning model that can accurately predict barley yield in Iran. To this end, the estimation model was trained using four machine learning techniques, including backpropagation neural network, decision tree, gaussian process regression, and K-nearest neighbor regression. The study also used the proposed model to investigate the correlation between the time intervals of the year and the location regarding the accuracy of yield prediction. It shows that the accuracy of the prediction is affected by location and time interval. However, the limitation that this model benefits from a relatively smaller set of features in the modeling process may have affected the prediction accuracy in the current setting.

So far as the characteristics of the above works relative to the focus areas of the current study are concerned, different approaches have covered the requirements in various ways and to a variety of extents. The image quality enhancement is carried out by processing the input image in a way that improves the distinctive features of the images, thus positively impacting the prediction results. Kamir et al. have explicitly addressed the issue of improving the input image data. To this end, a region of interest is determined from the dataset images, and within that region, all distorted pixels are masked and filled using linear temporal interpolation. Bian et al. improved the quality of multi-spectral UAV images used as input by employing a multi-stage process involving radiometric calibration of images, their empirical linear correction, and obtaining a high-resolution orthophoto of the crop. A similar method for image correction and enhancement was used by Meraj et al. to remove atmospheric errors. In addition, the images obtained in various scenes were mosaicked.

Optimization of the technique applied for feature extraction refers to studying the impact of various feature extraction techniques and using the best among the investigated methods for obtaining features for training the model. To this end, Paudel et al. [29,35] provided an approach to design features comprising physical meaning relative to their impact on crop development. However, other possibilities for feature development, e.g., the use of a more effective model for automatic feature extraction, have not been addressed. Similarly, Meroni et al. optimized the feature selection process using an approach that ensures minimum redundancy and maximum relevance, but without investigating the selected feature set’s impact on the estimation model’s overall performance. In contrast with most recent works, Meraj et al. have also performed the classification of acreage to be used for wheat based on the crop’s specific details with a unique spectral signature. The traditional machine learning approaches for yield estimation, in contrast with their deep learning-based counterparts, might be viewed as inherently lightweight and more suitable for deployment on small, embedded devices. However, none of the approaches has explicitly addressed the containment of the computational expense involved in the estimation using the proposed methods.

### 2.2. Deep Learning-Based Methods

Qiao et al. [25] used a combination of 3D convolutional and recurrent neural networks to predict wheat and corn yields from China. The proposed model is trained first on features obtained from the multi-spectral images, and then on the temporal data from the long time-series images. The study adopted two sensor datasets, i.e., the surface reflectance dataset (MOD09A1), and MODIS (moderate resolution imaging spectroradiometer) Annual Land Cover dataset (MYD11), acquired with the MODIS sensor. One of the prominent features of this work is its ability to handle multi-temporal and multi-spectral data simultaneously. YieldNet [26] is a deep learning framework that adopts transfer learning to make corn and soybean yield predictions for up to four months before the harvest in various counties of the United States.

The model uses a common feature extractor, which reduces the number of network parameters and reuses the common parameters for both crops, contributing to an increase in prediction efficiency. YieldNet could provide higher performance on different datasets related to yield performance and satellite images compared to the traditional machine learning models. Cotton provides the raw material for the cotton textile industry. It is one of the most important crops around the world. It is crucial to the industrial and agricultural economies of various countries. Xu et al. [40] estimated the cotton yield using time series UAV remote sensing data. They used a neural network based on the Bayesian regularization backpropagation to predict the cotton yield for both large-area and small-scale settings. Soybean is a good source of protein, fiber, and oil. Some studies have focused on the estimation of soybean yield. DeepYield [27] proposed to integrate the use of convolutional short-term memory (ConvLSTM) with a three-dimensional convolutional neural network (3D-CNN) to enhance the prediction accuracy of soybean yield in the US. We can see a significant contribution of this study to how it handles remote sensing images. Most existing approaches convert the spatial dimension of remote sensing images into histograms of pixel intensities. This results in the discarding of the spatial dimensions of the images.

In contrast, this model has utilized the spatial dimension to determine the important crop variables (e.g., soil properties and elevation), and thus improved the model’s forecasting ability. The performance of the proposed model was compared to other models, such as decision trees, CNN combined with the Gaussian process, and CNN-LSTM. DeepYield outperformed these techniques and each of the ConvLSTM and 3D-CNN models when those were used individually.

Regarding the focus areas addressed in the current study, Qiao et al. have comprehensively addressed the issue of input image quality enhancement. The irregular data from images taken from variously shaped fields are transformed into a cubic to achieve tractability by employing a dimension-transform technique. For this purpose, an optimal crop pixel threshold is determined, and all pixels lying exceedingly higher or lower than the threshold are considered noise, and are thus eliminated. Similarly, Khaki et al. developed an optimized set of dataset images by creating compact histogram representations of the sequences of multi-spectral images. Xu et al. provide the neural network model used for prediction with a fusion of the high-resolution images and the cotton bolls opening pixels extracted using a U-Net semantic segmentation network. Images are enhanced by time series data fusion. One of the distinctive features of this approach is the model’s simplicity, which is achieved by optimizing the input variables using sensitivity analysis.

Qiao et al. optimized the feature extraction by using a 3D CNN that jointly captures and fuses the spatial and spectral information found in both types of features. Then, a temporal dependency capturing module is used for temporal dependencies obtained from different multi-spectral images. The features obtained using this process are more comprehensive, and eventually improve the prediction performance. Khaki et al. implemented a CNN-based feature extractor to obtain relevant features from input data, and used it as a common backbone feature extractor to decrease the network parameters.

## 3. Methodology

**Figure 1:**
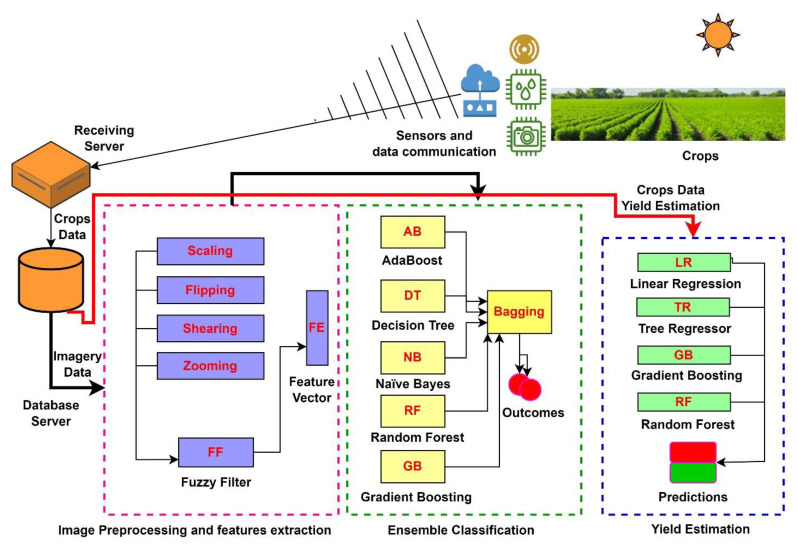
Block diagram of the proposed solution.

The agricultural field sensors, i.e., field surveillance cameras, temperature sensors, humidity sensors, fire-alert sensors, flood-warning sensors, and weather monitoring sensors, provide imagery and field-sensed data. These remote devices glimpse useful information about the status of different crops, estimate the crop yield, and notify about potential crop hazards. The remote visionary and sensory algorithms depend on the quality of images and field data for optimal crop classification and yield prediction. Figure 1 glimpses the general architecture of our proposed solution.

The enhancement of images is an essential step before feeding the inputs to deep learning algorithms for classifying different crops. The receiving server receives and archives the remotely captured images of crops under various parametric conditions. The database server contains multiple pools of acquired images as a sequence of time-series data. The architecture selects a pool of images and preprocesses for image classification. The preprocessed feature vector contains the features of images filtered image embedder. As a first instance, we identified the robust classifiers and achieved the classification outcomes with classification weights adjustment, so that our proposed ensemble classification method outperforms the weak classification methods on all images processed as per the time-stamped sequence of images from the image database server. The proposed architecture is equally supportive for embedded devices, where remote devices can adopt a lightweight classification algorithm, such as MobilenetV2. This can significantly reduce the processing time and overhead of a large pooled image.

**Figure 2:**
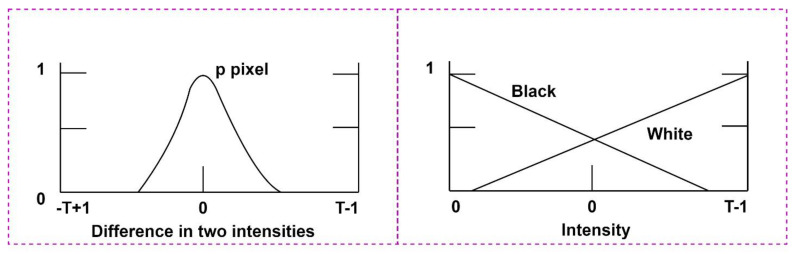
Intensity differences with the application of fuzzy rules.

We apply the fuzzy technique for spatial filtering for input image enhancement in the spatial domain based on neighborhood consideration. We take the neighborhood span of 3 × 3 by focusing central pixel intensity around all its neighboring dimensions. Let us consider p_1_, p_2_, p_3_, …, p_9_ image pixels in a 3 × 3 grid with corresponding intensity difference d_1_, d_2_, d_3_, …, d_9_. We calculate the intensity difference of p_i_, (for i ≤ 9) with its neighbors, and present the intensity variations following the following fuzzy rules. If pixels at corresponding locations shown are zero, then p_i_, (for i ≤ 9) is set to white; otherwise, black. The correspondence membership function is shown in Figure 2.

Figure 2 demonstrates the difference in intensities with the application of fuzzy rules at fuzzy space {0, Black, White}. The intensity level of “Black” gradually decreases within the total gray level span of 0 to T-1. Similarly, the intensity level of “White” gradually increases with the total gray level span of 0 to T-1. The fuzzy memberships provide a significant contour of images, later leading to viable extraction of image features.

To achieve the contrast enhancement of pooled images, we performed the necessary application of scaling, flipping, shearing, and zoom filters. Algorithm 1 describes the ensemble classification of pooled images, and Algorithm 2 depicts yield estimation.
**Algorithm 1:** Ensemble Classification of pooled images1:**Inputs:** Preprocessed feature vector **FE**2:**Outputs:** Classification outcomes ***C1***, and ***C2***3: *Let us us take a collection **P** = {P_1_, P_2_, P_3_, P_i_} of image vector space, where i ≤ **N***4:  *Let us apply scaling, flipping, shearing, and zooming filters to n images from collection **P**∀ n ≤ **N***5:  *Let us extract the features by applying image embedding to extract **F** = {F_1_, F_2_, F_3_, F_i_} features of images∀ i ≤ **N***6:   *Analyze Pi instances with features **FE** using AdaBoost classifier where each Pi in **P***7:   *Analyze Pi instances with features **FE** using the Decision tree classifier, each Pi in **P***8:   *Analyze Pi instances having features **FE** using Naïve Bayes classifier, each Pi in **P***9:   *Analyze Pi instances having features **FE** using Random Forest classifier, each Pi in **P***10:   *Analyze Pi instances with features **FE** using Logistic regression where each Pi in **P***11:   *Analyze the individual performance of all classifiers on Pi attributes of **P** for i ≤ **N***12:   *Output the classification as a result **Y** (Y ≤ 5) classifiers*13:  **End**14: **End**

The image embedder here convolves the image vector ***P*** in a series of convolution operations described **A**^P^ → **B**^P^ → **C**^Q^ → **D**^Q^ → …… **A**^Z^ → **B**^Z^ → **C**^T^. Let us consider vector **I** as an input image. The first layer of the embedder incorporates a weight **B** (a vector numerically applied to **I**, and the outcome serves as the input for the next layer). Similarly, we take vector **C^Q^** as an output of the first layer. Let us say the weight **D**^Q^ of the second layer, the convolution operation of **C^Q^** and **D^Q^** produces another vector, and the process keeps moving for a specific defined number of layers.
**Algorithm 2:** yield estimation1:**Inputs:** Preprocessed feature vector **F**2:**Outputs:** Estimation outcome ***E***3: *Let us take a collection **P** = {P_1_, P_2_, P_3_, P_i_} of field sensors data, where i ≤ **N***4:  *Let us apply preprocessing filters to n sensed data items from collection **P**∀ n ≤ **N***5:  *Let us extract the features of sensed data as vector **F** = {F_1_, F_2_, F_3_, F_i_}∀ i ≤ **N***6:   *Analyze Pi instances with features **F** using Linear Regressor where each Pi in **P***7:   *Analyze Pi instances with features **F** using GradientBoosting where each Pi in **P***8:   *Analyze Pi instances with features **F** using Tree Regressor where each Pi in **P***9:   *Analyze Pi instances with features **F** using Random Forest regressor, each Pi in **P***10:   *Analyze the individual performance of all estimators on Pi attributes of **P** for i ≤ **N***11:   *Output the estimation as an estimation vector E*12:  **End**13: **End**

Consider a pool of remotely collected images as a collection $P=\left\{P_{1},P_{2},\;P_{3},\;P_{4},\ldots .,P_{n}\right\},\;n\leq N$ of “n” images ϵ N images. The image-vector ***P*** of image space ***P*** is considered a “D” dimensional vector ∀ ***P*** ϵ R^D^. Moreover, let us take ***P*** ϵ R^XxYxZ^; each **X**, **Y**, and **Z** depict the row, column, and color vectors, respectively. We can further demonstrate these vectors with precise indices a, b, and c, ∀ 0 ≤ a ≤ D, 0 ≤ b ≤ D, and 0 < c ≤ 3.

1. If we represent the width and height of an image with “w” and “h”, the scaling filter provides a scaled image ***P′*** (w′, h′) ∀ (w′, h′) = T, where T is the maximum value as shown in Equation (4),

(4) $$(w^{\prime },\;h^{\prime })=\frac{T}{\max\left(w,\;h\right)}\left(w,\;h\right).$$

2. Correspondingly, the vertical and horizontal shearing defined for image vector ***P*** having coordinates p and q can be written as,

(5) $$\left(\begin{array}{c} p\prime \\ q\prime \end{array}\right)=\left(\begin{array}{c} p+mq\\ q \end{array}\right)=\left(\begin{array}{c} 1\;\;m\\ 0\;\;\;1 \end{array}\right)\left(\begin{array}{c} p\\ q \end{array}\right)$$

(6) $$\left(\begin{array}{c} p\prime \\ q^{\prime } \end{array}\right)=\left(\begin{array}{c} p\\ mx+q \end{array}\right)=\left(\begin{array}{c} 1\;\;0\\ m\;\;\;1 \end{array}\right)\left(\begin{array}{c} p\\ q \end{array}\right)$$

Equations (5) and (6) present the horizontal and vertical shearing of image vector ***P***, thus relocating the required data points in shearing the image ***P***.

3. Next, we apply the zooming to image vector ***P*** to seek a zoomed image vector ***P*’**, such that ***P*’** is nearly (r * 10%) of ***P***. The “r” here refers to a particular point of interest in image ***P*** having coordinates x and y. This zooming provides a zoomed vector of points (zx, zy) as a displacement of “r”. Since zooming is a trial-and-error-based process requiring the best compromise, the enhanced image vector should serve the desired purpose.
4. Further, the horizontal flip of an image vector ***P*** having coordinates x and y gives us an image ***P*’** with coordinates x’ and y’ ∀ x’ = width (vector ***I***) − x − 1 while y’ = y.

Repeat (1): x in range (width of vector ***P***)
Repeat (2): y in range (height of vector ***P***)
x′ = width (vector ***P***) − x − 1
End Repeat (2):

We identified the potentially strong classification methods to process our pooled images. The ensembled architecture contains Logistic Regression for describing the relationship between the predictor and target variables. The target variable is a multi-classified variable having values 1 to 6 referring to different crops (shown in Equations (7)–(12)).

(7) $$P\left(y=1|x\right)=h_{\theta }\left(x\right)=\frac{1}{1+\exp\left(-\theta^{T}x\right)}\equiv \sigma \left(\theta^{T}x\right)$$

(8) $$P\left(y=2|x\right)=h_{\theta }\left(x\right)=\frac{1}{1+\exp\left(-\theta^{T}x\right)}\equiv \sigma \left(\theta^{T}x\right)$$

(9) $$P\left(y=3|x\right)=h_{\theta }\left(x\right)=\frac{1}{1+\exp\left(-\theta^{T}x\right)}\equiv \sigma \left(\theta^{T}x\right)$$

(10) $$P\left(y=4|x\right)=h_{\theta }\left(x\right)=\frac{1}{1+\exp\left(-\theta^{T}x\right)}\equiv \sigma \left(\theta^{T}x\right)$$

(11) $$P\left(y=5|x\right)=h_{\theta }\left(x\right)=\frac{1}{1+\exp\left(-\theta^{T}x\right)}\equiv \sigma \left(\theta^{T}x\right)$$

(12) $$P\left(y=6|x\right)=h_{\theta }\left(x\right)=\frac{1}{1+\exp\left(-\theta^{T}x\right)}\equiv \sigma \left(\theta^{T}x\right)$$

To keep the value of $\theta^{T}x$ within 1 and 6, we use the Sigmoid function. The value of 1 is adjusted such that $P\;(y=1|x)=h_{\theta }\;\left(x\right)$ is large when x belongs to 1, else small when x belongs to other values. The sigmoid function (Equation (13)) is,

(13) $$\sigma \left(t\right)=\frac{1}{\left(1+e^{-t}\right)\;}$$

Further, we identified the support vector machine as another potential contributor to enhance the accuracy of the ensemble classification method. The basic idea was to find a hyperplane $\vec{\omega }$ that not only separates the data point from different classes, but also has a margin as large as possible. Equation (14) explains the parameters for creating a hyperplane,

(14) $$\vec{\omega }=\underset{j}{\sum }\alpha_{j}y_{j}\vec{d_{j}}$$

where, $y_{j}$ ∈ {1, 2, 3, 4, 5, 6}, which is the correct class of document d_j_ corresponding to the image class of the respective crop, and $\alpha_{j}$ is derived by solving dual optimization problems.

## 4. Results and Discussion

### 4.1. Results

We adopted publicly available datasets for yield prediction from the Food and Agriculture Organization (FAO) of the United Nations (available online http://www.FAO.org, accessed on 5 December 2022) and the Word Bank DataBank (available online https://databank.worldbank.org/home.aspx, accessed on 5 December 2022). We also augmented the imagery datasets before preprocessing to achieve an unbiased and fair classification between different crop types. We covered the diversity of various crops, for instance, jute, maize, rice, sugarcane, and wheat, for classification aspects, and flaxseed, lentils, rice, sugarcane, and wheat for yield estimation. This study investigated the performance of different classifiers using the following evaluation metrics (shown in Equations (15)–(18)).

True Positive (TP): The outcome of the model when the model correctly predicts the positive class

False Positive (FP): The outcome of the model when the model incorrectly predicts the positive class

True Negative (TN): The outcome of the model when the model correctly predicts the negative class

False Negative (FN): The outcome of the model when the model incorrectly predicts the negative class

(a) Accuracy: The proportion of true results to the total number of cases examined,
  (15) $$\mathrm{Classification}\;\mathrm{Accuracy}\;\left(\mathrm{CA}\right)=\frac{\mathrm{TP}+\mathrm{TN}}{\mathrm{TP}+\mathrm{FP}+\mathrm{FN}+\mathrm{TN}}\times 100\%$$
(b) Precision: Determines the proportion of predicted positives to be truly positive,
  (16) $$\mathrm{Precision}=\frac{\mathrm{TP}}{\mathrm{TP}+\mathrm{FP}}\times 100\%$$
(c) Recall: Identifies the proportion of actual positives correctly classified,
  (17) $$\mathrm{Recall}=\frac{\mathrm{TP}}{\mathrm{TP}+\mathrm{FN}}\times 100\%$$
(d) AUC: Indicates how well the probabilities from the positive classes are separated from the negative classes.
(e) F1-Score: The overall performance of the model is measured
  (18) $$F1-\mathrm{Score}=\frac{2\mathrm{TP}}{2\mathrm{TP}+\mathrm{FP}+\mathrm{FN}}\times 100\%$$

**Figure 3:**
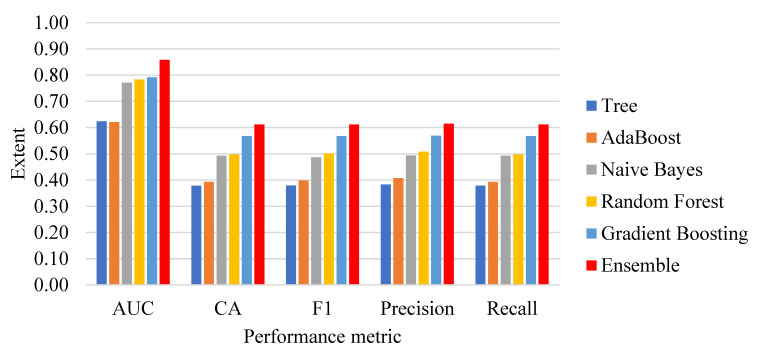
Performance analysis of classification methods.

Figure 3 depicts the performance achievement of different classification methods. The proposed ensemble classification method outperforms the other individual classifiers, since the study identified the strong classifiers that boosted the performance of the ensemble method. Since AUC is a good measure that provides a fair ratio between sensitivity and specificity, the area under the curve shows larger peaks of the ensemble classification method. Similarly, the F1 score is also an unbiased harmonic evaluation metric, and the ensemble method outperforms the other classifiers significantly.

**Figure 4:**
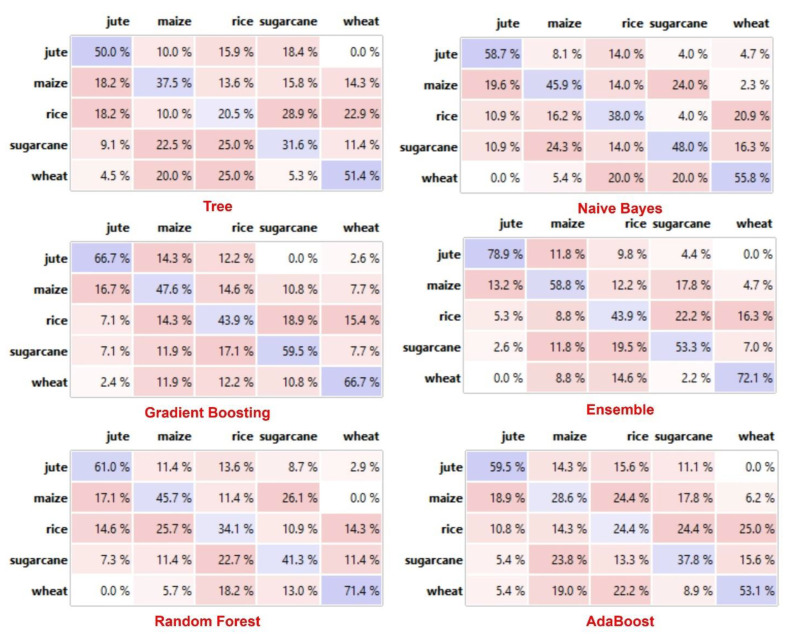
Confusion metric analysis of classifiers.

Figure 4 presents the comparative analysis of classification methods based on confusion Metrix. The ensemble method correctly identified the crops as compared to other methods. For instance, the algorithm classified the jute crop with 78% accuracy and maize with 59% accuracy, while the classification of rice, sugarcane, and wheat remained at 44%, 53%, and 72%, respectively. These performance measures are significantly better than the individual classification methods for all crop types. We identified the poor performance of the decision tree classification method that depicted only 50% accuracy for jute, 37% for maize, 20% for rice, 32% for sugarcane, and 51% for the wheat crop.

**Figure 5:**
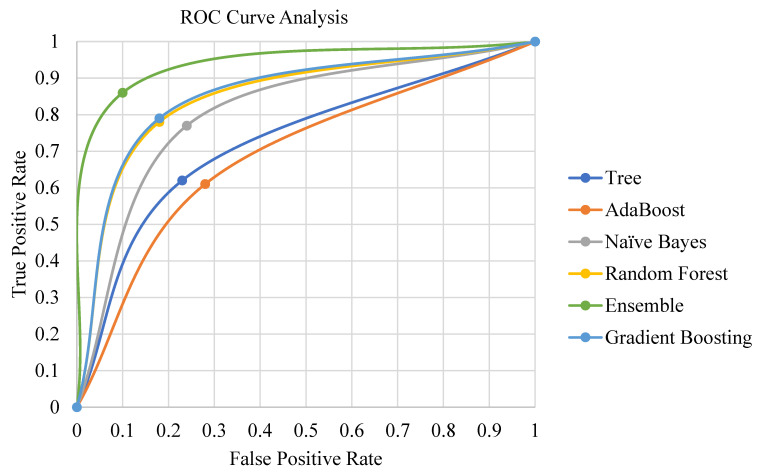
ROC curve analysis.

Table 2 shows that the ensemble method has a pronounced degree of significance compared to decision trees, random forests, Naïve Bayes, gradient boosting, and AdaBoost classification methods. There is a slight difference in the classification performance of gradient boost and random forest classifiers. The decision tree classification method underperforms compared to other classification methods. The ROC curve in Figure 5 also demonstrates the significant performance of the ensemble classification method.

Table 3 presents the year-wise yield estimation of different crops under this study. We covered the yield years 2017 to 2021 relevant to the available data of crops shown in the table. We considered a random forest, gradient boosting, linear regression, and tree regressors to predict yield estimates. We discuss the yield estimation in the subsequent discussion section.

### 4.2. Discussion

Modern precision tools are the backbone of industry 4.0-based agriculture growth, adding considerable value to countries’ gross domestic product (GDP). We proposed a hybrid ensemble method investigating the limitations of existing similar works. The convolution operation of convolution neural networks (CNNs) is critical in determining strong feature vectors of remote sensory images and impacting classification methods’ performance. The classification methods become computationally intensive for larger sets of high-dimensional data to achieve better performance. The proposed hybrid ensemble method exploited VGG-16, among several other available state-of-the-art (SOTA) feature extraction methods. We compared the performance of VGG-16, Inception-V3, DeepLoc, SqueezeNet, and VGG-19 on a considerably larger set of remote sensory augmented images of different crop types. The component variance and cumulative variance in terms of proportion of variance in principle component analysis are given below.

**Figure 6:**
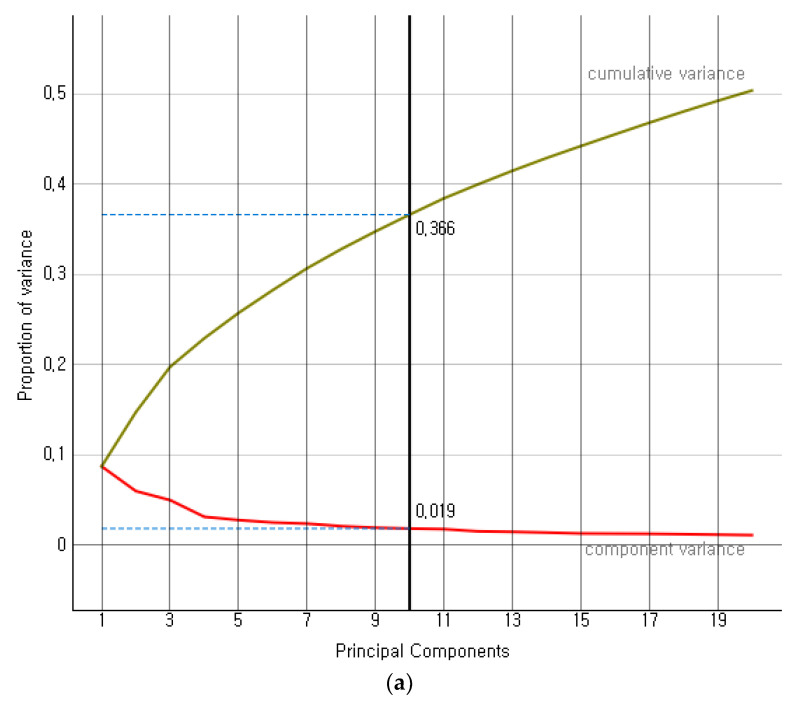

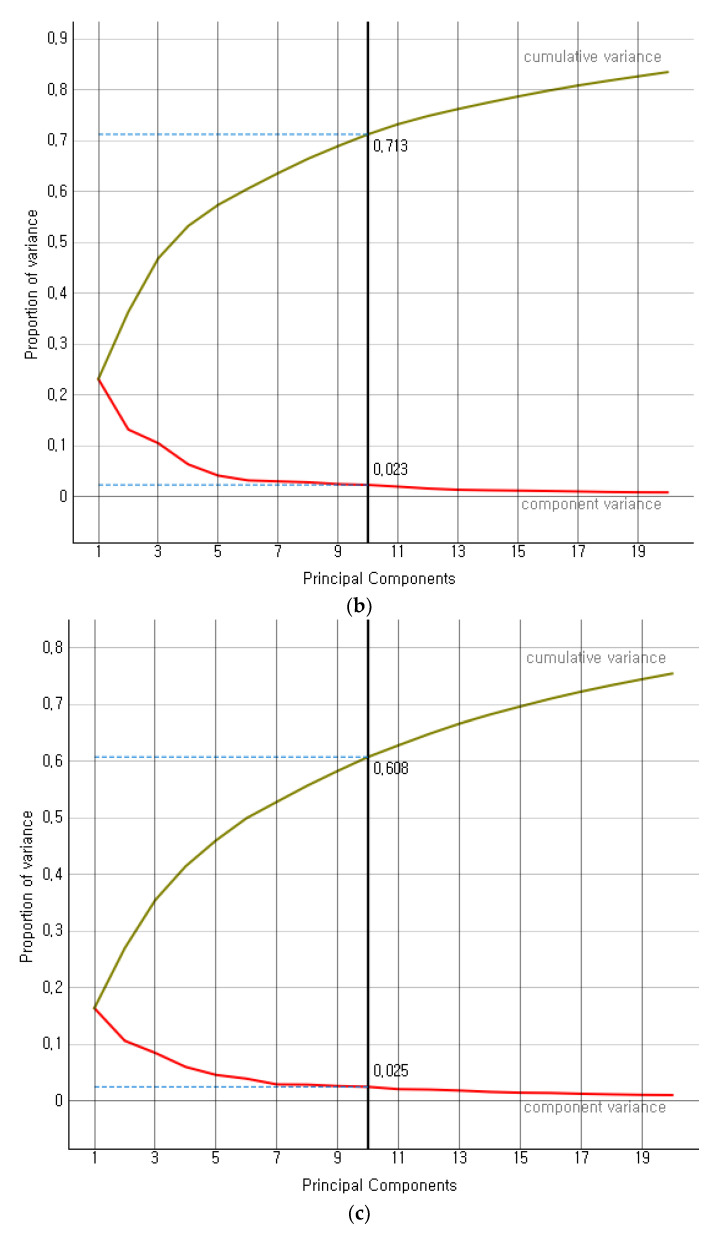

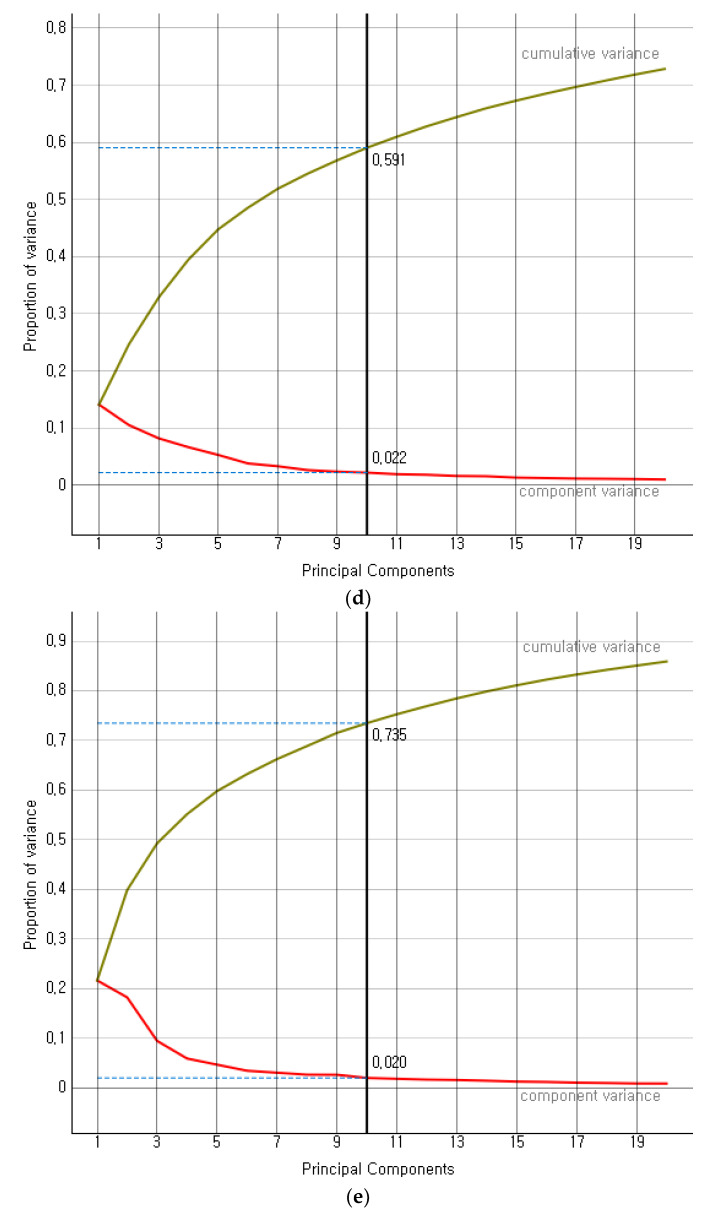
(**a**) Proportion of variance for Inception V3, (**b**) Proportion of variance for SqueezeNet, (**c**) Proportion of variance for VGG-16, (**d**) Proportion of variance for VGG-19, (**e**) Proportion of variance for DeepLoc.

Figure 6 presents the proportion of variance of the first ten principal components of four SOTA feature extraction methods. The Inception-V3, VGG-16, VGG-19, DeepLoc, and SqueezeNet depicted an explained variance of 36%, 60%, 59%, 73%, and 71%, respectively, on the first 20 normalized variables. The VGG-16’s explained variance, i.e., 60%, fell close to the mean-variance (59.8%) of the SOTA methods in consideration. Further, we simulated the performance of SOTA convolution methods on five larger random datasets of remote sensory images. The cumulative performance analysis is given below.

**Figure 7:**
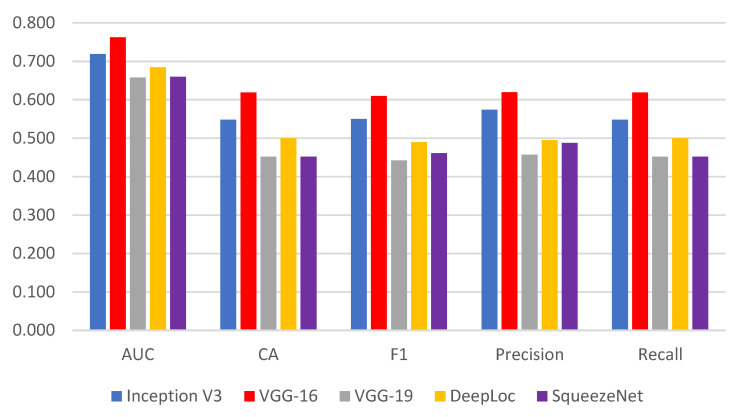
Performance of SOTA convolution methods.

Figure 7 presents the performance metric of the SOTA convolution methods. The VGG-16 described the best feature extraction on target data, achieving significant classification accuracy, the area under the curve, F1-score, precision, and recall. Based on this performance analysis, we employed VGG-16 as a prominent feature extractor for the proposed ensemble method.

It is well known that convolution neural networks significantly impact the performance of many state-of-the-art classification and prediction methods. The enhanced performance of our proposed ensemble method relies heavily on feature extraction methods. We identified VGG-16 as the best feature extractor in this problem domain. In addition, considering different use cases of CNNs, simulated on multiple sensory data, we evaluated the performance of VGG with image kernels. Although the performance of SOTA methods is architecture-dependent, the performance of VGG-16 was noted to be better with relatively faster training time. It ultimately helped to achieve better classification accuracy on a bunch of enriching weak classifiers (discussed in the proposed ensemble method).

**Figure 8:**
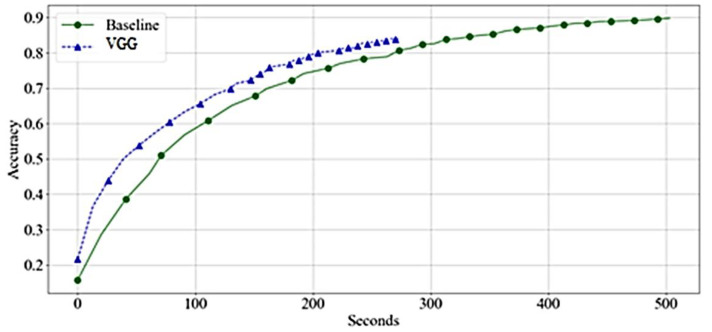
Accuracy by Time-VGGs—Training.

Figure 8 compares the needed time to achieve the accuracy of the network. The VGG was found significant, and trained faster than the baseline. The training time per epoch reduces significantly in the middle of the training.

**Figure 9:**
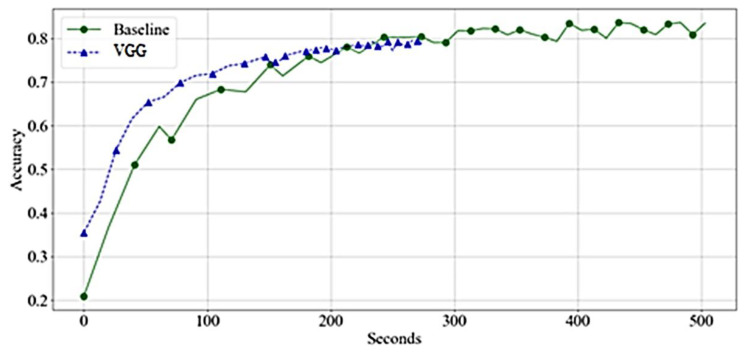
Accuracy by Time-VGGs—Validation.

Figure 9 shows that the VGG reaches high accuracy significantly faster than the baseline. However, the acceleration is due to faster training time per epoch rather than achieving higher accuracy with a lower number of epochs.

**Figure 10:**
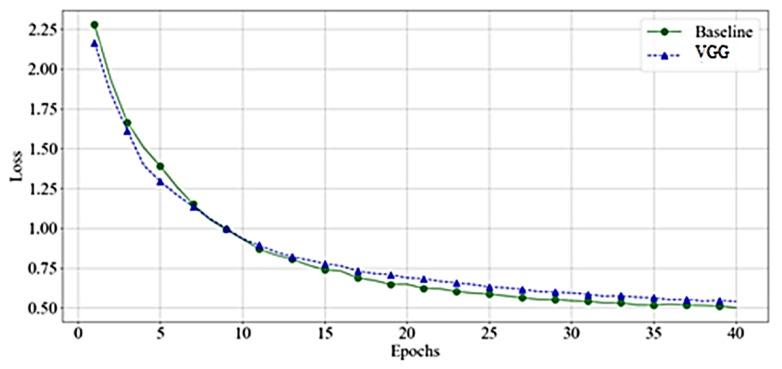
Loss Value-VGGs—Training.

**Figure 11:**
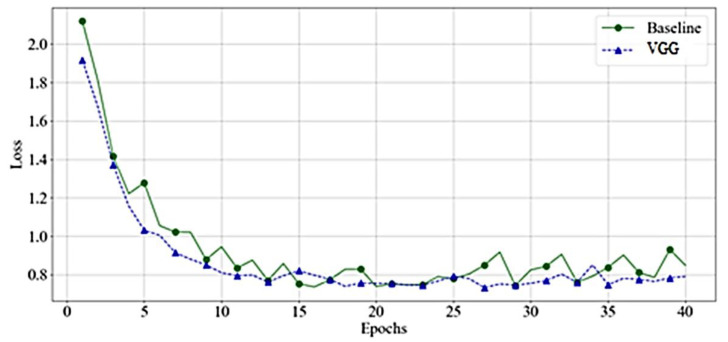
Loss Value-VGGs—Validation.

As shown in Figure 10, the VGG and baseline loss function values had almost identical behavior on training samples. However, as Figure 11 demonstrates, the baseline loss has more fluctuation in the case of the validation dataset.

**Figure 12:**
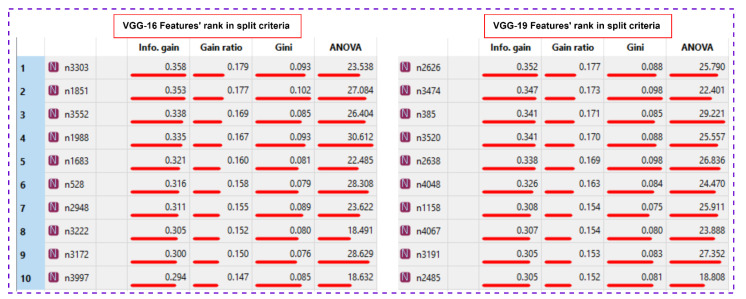
Features’ rank criteria of both networks.

Both networks have a smooth decrease in loss function with no signs of overfitting. However, the VGG had less fluctuation compared to the baseline. This behavior reappears in other experiments. Since we noticed a comparable performance of VGG-16, and VGG-19, Figure 12 presents the features’ rank criteria of both networks. We considered information gain, gain-ratio, Gini-index, and ANOVA as good qualifiers to identify the strongest features of data. The qualifiers have higher to lower values, corresponding to strongest to weakest features. Since the cumulative component variance VGG-16 was better than VGG-19, we employed VGG-16 as the best convolution network for feature extraction of remote sensory data.

Further, we considered random forest, gradient boosting, linear regression, and tree regressor to predict yield estimates, as shown in Table 4.

**Figure 13:**
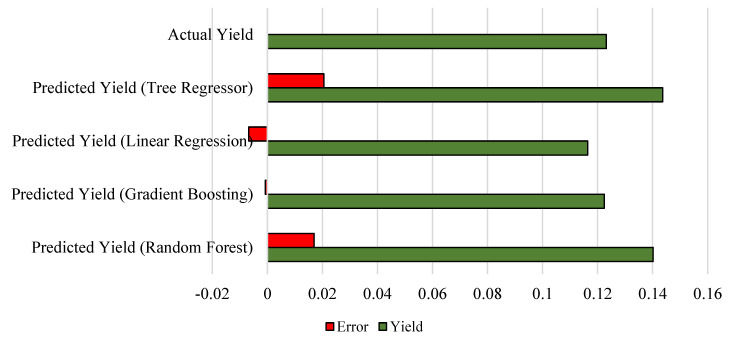
Analysis of yield versus residual.

We normalized the yield data to fit well for prediction algorithms. The normalized actual yield is 0.123177. We can see that the gradient boosting algorithm outperforms the other predictors and achieves negligible residual compared with the actual yield. Figure 13 depicts the yield and error terms.

Further, we performed the analysis of variance (ANOVA) test on our simulated results (including both SOTA and machine learning methods), and we achieved the following outcomes.

In Table 5, chosen on a 95% confidence interval (α = 0.05), the F value is 3.36, which is significantly larger than the F-critical value of 2.62, achieving a *p*-value of 0.01, which is significantly smaller than 0.05.

Similarly, we performed the ANOVA test on the outcomes of SOTA methods used for feature extraction of remote sensory images. Here are the findings.

In Table 6, chosen on 95% confidence interval (α = 0.05), the F value is 3.07, which is significantly larger than the F-critical value of 2.866, achieving a *p*-value of 0.03, significantly smaller than 0.05.

## 5. Conclusions

Remote data analysis is immensely important for today’s precision agriculture. This study presented a fuzzy hybrid ensembled classification and estimation of crop yields using remote sensory data. The proposed architecture enhanced the pooled images with a fuzzy neighborhood filter and image preprocessing. The study identified the optimal weights of the strongest candidate classifiers for the ensembled classification method adopting the bagging strategy. The study achieved unbiased classification on augmented imagery datasets for jute, maize, rice, sugarcane, and wheat. Considering the diversity of crops, the study exploited yield estimation of flaxseed, lentils, rice, sugarcane, and wheat on publicly available datasets. The ensemble method outperformed the individual classification methods for crop type classification on an average of 13% and 24%, compared to gradient boosting and decision tree methods, respectively. Similarly, we observed that the gradient boosting predictors outperformed the multivariate regressor, random forest, and tree regressor, with a comparatively lower mean square error value on yield years 2017 to 2021. Prospectively, the proposed architecture can be used for embedded devices with lightweight CNN, i.e., MobilenetV2. This can greatly reduce the processing time and overhead of a large set of pooled images.

## Figures and Tables

**Table 1:** Summary of works related to crop yield estimation using machine learning methods.

Reference	Feature/Model	Crop(s)	Focus Areas			
			Image Quality Enhancement	Optimization of Feature Extraction	Joint Crop Classification and Yield Estimation	Suitability for Small, Embedded Devices
**Traditional Methods**						
Paudel et al. [29]	WOFOST features, RR, kNN, SVM, gradient boosted DT	Soft wheat, spring barley, sunflower, sugar beet, potatoes	✕	✓	✕	~
Meroni et al. [30]	LASSO, RF, MLP, SVR	Barley, soft wheat and durum wheat	✕	✓	✕	~
Paudel et al. [35]	WOFOST features, RR, kNN, SVM, gradient boosted DT	Wheat, barley, sunflower, grain maize, sugar beets and potatoes	✕	✓	✕	~
Zhou et al. [31]	RF, SVM, and least absolute shrinkage and selection operator	Wheat	✕	✕	✕	~
Kamir et al. [36]	RF, XGBoost, Cubist, MLP, SVR, GPR, kNN	Wheat	✓	✕	✕	~
Bian et al. [37]	GPR, SVR, RF	Wheat	✓	✕	✕	~
Cao et al. [38]	XGBoost, random forest, and support vector regression	Wheat	✕	✕	✕	~
Meraj et al. [28]	RF, SVM, CASA	Wheat	✓	✕	✓	~
Sharifi [39]	BP-NN, DT, GPR	Barley	✕	✕	✕	~
**Deep Learning-Based Methods**						
Qiao et al. [25]	Multi-temporal and multi-spectral data, 3D CNN + RNN	Wheat, corn	✓	✓	✕	✕
Khaki et al. [26]	3D histograms, CNN,	Corn, soybean	✓	✓	✕	✕
Xu et al. [40]	Fusion of images, BP-NN	Cotton	✓	✕	✕	✕
Gavahi et al. [27]	ConvLSTM + 3D-CNN	Soybean	✓	✓	✕	✕

✓: characteristic explicitly addressed; ~: characteristic implicitly addressed; ✕: characteristic not addressed; RR: Ridge Regression; RF: random forest; SVM: support vector machine; LASSO: least absolute shrinkage and selection operator; BP-NN: backpropagation neural network; DT: decision tree; GPR: gaussian process regression; CNN: convolutional neural network; RNN: recurrent neural network; SVR: support vector regression; CASA: Carnegie-Ames-Stanford Approach; MLP: multi-layer perceptron.

**Table 2:** Cross-tabulation analysis of classifiers.

	Decision Tree	Random Forest	Naive Bayes	Gradient Boosting	Ensemble Classifier	AdaBoost
Decision Tree		0.00	0.00	0.00	0.00	0.54
Random Forest	1.00		0.75	0.48	0.03	0.99
Naive Bayes	1.00	0.24		0.28	0.00	0.99
Gradient Boosting	0.99	0.51	0.71		0.00	0.99
Ensemble Classifier	1.00	0.94	0.99	0.99		1.00
AdaBoost	0.54	0.00	0.00	0.00	0.00	

**Table 3:** Yield estimation of different crops.

Crop Type	Year					Grand Total
	2017	2018	2019	2020	2021	
**Flaxseed**						
Acres harvested	2,885,033	1,013,800	1,248,000	1,442,000	1,439,000	8,027,833
Acres planted	1,683,700	1,512,400	2,148,000	2,060,000	2,420,000	9,824,100
Yield (BU/acre)	500.3	607.6	56.8	419.8	143.1	1727.6
**Lentils**						
Acres harvested	10,890,453	5,660,200	3,369,800	3,353,000	4,018,000	27,291,453
Acres planted	6,227,300	7,610,600	4,792,000	4,464,000	5,880,000	28,973,900
Yield (LB/acre)	36,105	51,100	35,218	41,239	17,669	181,331
**Rice**						
Acres harvested	70,529,219	39,990,500	35,337800	38,378,000	32,922,000	217,157,519
Acres planted	27,415,200	28,450,000	26,232,900	26,718,000	23,420,000	132,236,100
Yield (LB/acre)	906,294	867899	828,796	807,735	719,627	4,130,351
**Sugarcane**						
Acres harvested	255,981	80,200	85,800	88,400	93,800	604,181
Yield (tons/acre)	875.4	874.4	888.3	860.3	857.4	4355.8
**Wheat**						
Acres harvested	779,272,739	237,493,000	228,408,000	220,290,000	226,698,000	1,692,161,739
Acres planted	184,222,000	285,950,000	273,696,000	266,710,000	279,682,000	1,290,260,000
Yield (BU/acre)	2658.4	2627.2	2360.1	2330.8	2357.8	12,334.3

**Table 4:** Performance analysis of yield predictors.

Predictors	Yield (Normalized)	Error
Predicted Yield—Random Forest	0.140170633	0.017
Predicted Yield—Gradient Boosting	0.122418296	−0.001
Predicted Yield—Linear Regression	0.116406755	−0.007
Predicted Yield—Tree Regressor	0.143707889	0.021
Actual Yield	0.123177597	

**Table 5:** ANOVA: Statistical significance of outcomes of classification methods (α = 0.05).

Groups	Count	Sum	Average	Variance		
Tree	5	2.1420382	0.428407632	0.01194		
AdaBoost	5	2.2122979	0.442459586	0.00997		
Naive Bayes	5	2.7361336	0.547226728	0.01564		
Random Forest	5	2.7876549	0.55753097	0.01599		
Gradient Boosting	5	3.0632562	0.612651232	0.01006		
Ensemble	5	3.3086892	0.661737844	0.0120		
ANOVA						
Source of Variation	SS	df	MS	F	*p*-value	F crit
Between Groups	0.212041	5	0.04240814	3.36323	0.01921	2.620
Within Groups	0.302624	24	0.012609346			
Total	0.514665	29				

**Table 6:** ANOVA: Statistical significance of outcomes of feature extraction methods (α = 0.05).

*Groups*	*Count*	*Sum*	*Average*	*Variance*		
Inception V3	5	2.939	0.5878	0.0055		
VGG-16	5	3.23	0.646	0.004222		
VGG-19	5	2.461	0.4922	0.00862		
DeepLoc	5	2.67	0.534	0.007143		
SqueezeNet	5	2.513	0.5026	0.00796		
ANOVA						
*Source of Variation*	*SS*	*df*	*MS*	***F***	***p-value***	***F crit***
Between Groups	0.08228344	4	0.020571	**3.0754**	**0.03981586**	**2.866081**
Within Groups	0.1337768	20	0.006689			
Total	0.21606024	24				

## Data Availability

Not applicable.

## References

[1] Gaigbe-Togbe V., Bassarsky L., Gu D., Spoorenberg T., Zeifman L. (2022). World Population Prospects. https://www.un.org/development/desa/pd/sites/www.un.org.development.desa.pd/files/wpp2022_summary_of_results.pdf.

[2] Nodin M.N., Mustafa Z., Hussain S.I. (2022). Assessing rice production efficiency for food security policy planning in Malaysia: A non-parametric bootstrap data envelopment analysis approach. Food Policy.

[3] van der Berg S., Patel L., Bridgman G. (2022). Food insecurity in South Africa: Evidence from NIDS-CRAM wave 5. Dev. S. Afr..

[4] Al-Khateeb S.A., Hussain A., Lange S., Almutari M.M., Schneider F. (2021). Battling Food Losses and Waste in Saudi Arabia: Mobilizing Regional Efforts and Blending Indigenous Knowledge to Address Global Food Security Challenges. Sustainability.

[5] Government of Saudi Arabia (2020). Vision 2030 Kingdom of Saudi Arabia. https://vision2030.gov.sa/download/file/fid/417.

[6] Mu’azu N.D., Blaisi N.I., Naji A.A., Abdel-Magid I.M., AlQahtany A. (2019). Food waste management current practices and sustainable future approaches: A Saudi Arabian perspectives. J. Mater. Cycles Waste Manag..

[7] Alshabanat Z., Alkhorayef A., Ben Haddad H., Mezghani I., Gouider A., Tlili A., Allouche M.A., Gannouni K.A. (2021). Quantifying Food Loss and Waste in Saudi Arabia. Sustainability.

[8] Baig M.B., Gorski I., Neff R.A. (2019). Understanding and addressing waste of food in the Kingdom of Saudi Arabia. Saudi J. Biol. Sci..

[9] Ilyas Q.M., Ahmad M. (2020). Smart farming: An enhanced pursuit of sustainable remote livestock tracking and geofencing using IoT and GPRS. Wirel. Commun. Mob. Comput..

[10] Chlingaryan A., Sukkarieh S., Whelan B. (2018). Machine learning approaches for crop yield prediction and nitrogen status estimation in precision agriculture: A review. Comput. Electron. Agric..

[11] Wang A.X., Tran C., Desai N., Lobell D., Ermon S. Deep transfer learning for crop yield prediction with remote sensing data. Proceedings of the 1st ACM SIGCAS Conference on Computing and Sustainable Societies, COMPASS 2018.

[12] Sakamoto T., Gitelson A.A., Arkebauer T.J. (2013). MODIS-based corn grain yield estimation model incorporating crop phenology information. Remote Sens. Environ..

[13] Kogan F.N. (1995). Application of vegetation index and brightness temperature for drought detection. Adv. Sp. Res..

[14] Gitelson A.A. (2004). Wide Dynamic Range Vegetation Index for Remote Quantification of Biophysical Characteristics of Vegetation. J. Plant Physiol..

[15] Xue J., Su B. (2017). Significant remote sensing vegetation indices: A review of developments and applications. J. Sens..

[16] Tantalaki N., Souravlas S., Roumeliotis M. (2019). Data-driven decision making in precision agriculture: The rise of big data in agricultural systems. J. Agric. Food Inf..

[17] Akhter R., Sofi S.A. (2022). Precision agriculture using IoT data analytics and machine learning. J. King Saud Univ. Inf. Sci..

[18] Bu F., Wang X. (2019). A smart agriculture IoT system based on deep reinforcement learning. Futur. Gener. Comput. Syst..

[19] Magomadov V.S. (2019). Deep learning and its role in smart agriculture. J. Phys. Conf. Ser..

[20] Shafi U., Mumtaz R., García-Nieto J., Hassan S.A., Zaidi S.A.R., Iqbal N. (2019). Precision agriculture techniques and practices: From considerations to applications. Sensors.

[21] Tsouros D.C., Bibi S., Sarigiannidis P.G. (2019). A review on UAV-based applications for precision agriculture. Informatics.

[22] Wang A., Zhang W., Wei X. (2019). A review on weed detection using ground-based machine vision and image processing techniques. Comput. Electron. Agric..

[23] Li P., He D., Qiao Y., Yang C. (2013). An application of soft sets in weed identification. Am. Soc. Agric. Biol. Eng. Annu. Int. Meet..

[24] Bashar D.A. (2019). Survey on Evolving Deep Learning Neural Network Architectures. J. Artif. Intell. Capsul. Networks.

[25] Qiao M., He X., Cheng X., Li P., Luo H., Zhang L., Tian Z. (2021). Crop yield prediction from multi-spectral, multi-temporal remotely sensed imagery using recurrent 3D convolutional neural networks. Int. J. Appl. Earth Obs. Geoinf..

[26] Khaki S., Pham H., Wang L. (2021). Simultaneous corn and soybean yield prediction from remote sensing data using deep transfer learning. Sci. Rep..

[27] Gavahi K., Abbaszadeh P., Moradkhani H. (2021). DeepYield: A combined convolutional neural network with long short-term memory for crop yield forecasting. Expert Syst. Appl..

[28] Meraj G., Kanga S., Ambadkar A., Kumar P., Singh S.K., Farooq M., Johnson B.A., Rai A., Sahu N. (2022). Assessing the Yield of Wheat Using Satellite Remote Sensing-Based Machine Learning Algorithms and Simulation Modeling. Remote Sens..

[29] Paudel D., Boogaard H., de Wit A., Janssen S., Osinga S., Pylianidis C., Athanasiadis I.N. (2021). Machine learning for large-scale crop yield forecasting. Agric. Syst..

[30] Meroni M., Waldner F., Seguini L., Kerdiles H., Rembold F. (2021). Yield forecasting with machine learning and small data: What gains for grains?. Agric. For. Meteorol..

[31] Zhou W., Liu Y., Ata-Ul-Karim S.T., Ge Q., Li X., Xiao J. (2022). Integrating climate and satellite remote sensing data for predicting county-level wheat yield in China using machine learning methods. Int. J. Appl. Earth Obs. Geoinf..

[32] Oikonomidis A., Catal C., Kassahun A. (2022). Deep learning for crop yield prediction: A systematic literature review. N. Z. J. Crop Hortic. Sci..

[33] Rashid M., Bari B.S., Yusup Y., Kamaruddin M.A., Khan N. (2021). A Comprehensive Review of Crop Yield Prediction Using Machine Learning Approaches with Special Emphasis on Palm Oil Yield Prediction. IEEE Access.

[34] Muruganantham P., Wibowo S., Grandhi S., Samrat N.H., Islam N. (2022). A Systematic Literature Review on Crop Yield Prediction with Deep Learning and Remote Sensing. Remote Sens..

[35] Paudel D., Boogaard H., de Wit A., van der Velde M., Claverie M., Nisini L., Janssen S., Osinga S., Athanasiadis I.N. (2022). Machine learning for regional crop yield forecasting in Europe. Field Crop. Res..

[36] Kamir E., Waldner F., Hochman Z. (2020). Estimating wheat yields in Australia using climate records, satellite image time series and machine learning methods. ISPRS J. Photogramm. Remote Sens..

[37] Bian C., Shi H., Wu S., Zhang K., Wei M., Zhao Y., Sun Y., Zhuang H., Zhang X., Chen S. (2022). Prediction of Field-Scale Wheat Yield Using Machine Learning Method and Multi-Spectral UAV Data. Remote Sens..

[38] Cao J., Wang H., Li J., Tian Q., Niyogi D. (2022). Improving the Forecasting of Winter Wheat Yields in Northern China with Machine Learning–Dynamical Hybrid Subseasonal-to-Seasonal Ensemble Prediction. Remote Sens..

[39] Sharifi A. (2021). Yield prediction with machine learning algorithms and satellite images. J. Sci. Food Agric..

[40] Xu W., Chen P., Zhan Y., Chen S., Zhang L., Lan Y. (2021). Cotton yield estimation model based on machine learning using time series UAV remote sensing data. Int. J. Appl. Earth Obs. Geoinf..

